# Transcriptomic analysis reveals importance of ROS and phytohormones in response to short-term salinity stress in *Populus tomentosa*

**DOI:** 10.3389/fpls.2015.00678

**Published:** 2015-09-15

**Authors:** Lingyu Zheng, Yu Meng, Jing Ma, Xiulian Zhao, Tielong Cheng, Jing Ji, Ermei Chang, Chen Meng, Nan Deng, Lanzhen Chen, Shengqing Shi, Zeping Jiang

**Affiliations:** ^1^State Key Laboratory of Tree Genetics and Breeding, Research Institute of Forestry, Chinese Academy of ForestryBeijing, China; ^2^College of Landscape and Travel, Agricultural University of HebeiBaoding, China; ^3^College of Biology and the Environment, Nanjing Forestry UniversityNanjing, China; ^4^Chair of Proteomics and Bioanalytics, Technische Universität MünchenFreising, Germany; ^5^Institute of Apicultural Research, Chinese Academy of Agricultural SciencesBeijing, China; ^6^Risk Assessment Laboratory for Bee Products, Quality and Safety of Ministry of AgricultureBeijing, China

**Keywords:** *Populus tomentosa*, salt stress, transcriptomic analysis, differentially expressed genes, H_2_O_2_, hormone

## Abstract

*Populus tomentosa* (Chinese white poplar) is well adapted to various extreme environments, and is considered an important species to study the effects of salinity stress on poplar trees. To decipher the mechanism of poplar's rapid response to short-term salinity stress, we firstly detected the changes in H_2_O_2_ and hormone, and then profiled the gene expression pattern of 10-week-old seedling roots treated with 200 mM NaCl for 0, 6, 12, and 24 h (h) by RNA-seq on the Illumina-Solexa platform. Physiological determination showed that the significant increase in H_2_O_2_ began at 6 h, while that in hormone ABA was at 24 h, under salt stress. Compared with controls (0 h), 3991, 4603, and 4903 genes were up regulated, and 1408, 2206, and 3461 genes were down regulated (adjusted *P* ≤ 0.05 and |log2Ratio|≥1) at 6, 12, and 24 h time points, respectively. The Gene Ontology (GO) and Kyoto Encyclopedia of Genes and Genomes (KEGG) pathway annotation revealed that the differentially expressed genes (DEGs) were highly enriched in hormone- and reactive oxygen species-related biological processes, including “response to oxidative stress or abiotic stimulus,” “peroxidase activity,” “regulation of transcription,” “hormone synthetic and metabolic process,” “hormone signal transduction,” “antioxidant activity,” and “transcription factor activity.” Moreover, K-means clustering demonstrated that DEGs (total RPKM value>12 from four time points) could be categorized into four kinds of expression trends: quick up/down over 6 or 12 h, and slow up/down over 24 h. Of these, DEGs involved in H_2_O_2_- and hormone- producing and signal-related genes were further enriched in this analysis, which indicated that the two kinds of small molecules, hormones and H_2_O_2_, play pivotal roles in the short-term salt stress response in poplar. This study provides a basis for future studies of the molecular adaptation of poplar and other tree species to salinity stress.

## Introduction

More than 800 million hectares of land throughout the world are affected by salt, which accounts for more than 6% of the world's total land area; thus, soil salinity is becoming one of the crucial environmental factors that limit plant growth and development (Munns and Tester, 2008; Golldack et al., 2014). Moreover, saline regions are rapidly expanding because of ongoing climate change. Therefore, there is an urgent imperative to create salt tolerant plants (Munns et al., 2012), which requires a comprehensive understanding of the plant's response mechanism in high salinity conditions (Bartels and Sunkar, 2005). As a severe adverse condition, salt stress can induce ionic stress and osmotic stress in plant cells, which further result in the accumulation of reactive oxygen species (ROS) (Pang and Wang, 2008; Miller et al., 2010). High ROS levels not only result in oxidative damage and programmed cell death, but also act as signal molecules (Miller et al., 2010; Petrov et al., 2015). To cope with the oxidative damage resulting from ROS, plants have developed a complex scavenging system, including enzymatic and non-enzymatic (antioxidants) systems (Pang and Wang, 2008). However, to survive under such conditions, plants have to perceive and respond to these stresses rapidly (Bohnert et al., 1995; Chinnusamy et al., 2006) via signal transduction pathways mediated by stress hormones, such as abscisic acid (ABA; Fujii et al., 2009; Bhaskara et al., 2012; Chater et al., 2014) and ethylene (Wang et al., 2002; Merchante et al., 2013); other hormones, such as brassinosteroids (BRs; Cui et al., 2011); and small molecules, such as H_2_O_2_ (Verslues et al., 2007; Pang and Wang, 2008; Shi et al., 2010; Steffens et al., 2013). These responses lead to adaptive or morphological changes through activating complex regulatory networks that control global gene expression, protein modification and metabolite composition (Wang et al., 2002; Urano et al., 2010; Bhaskara et al., 2012; Merchante et al., 2013; Chater et al., 2014). Moreover, studies have demonstrated that H_2_O_2_ plays a critical role in mediating cross talk between hormones in model grass plants, such as *Arabidopsis* (He et al., 2012; Chen et al., 2014) and tomato (Zhou et al., 2014). However, the stress-specific responses, including ROS and phytohormone, remain unclear in woody plants or forest trees.

The mechanisms of stress responses in woody plants are also a challenging field of study because trees are long-lived and are constantly exposed to environmental changes. Poplar (*Populus*) is one of the most commonly used model woody plants, known for its fast growth rates among temperate trees (Taylor, 2002). It has evolved sophisticated systems to respond to abiotic stresses during its long life spans (Harfouche et al., 2014). The completion of the poplar (*P. trichocarpa*) genome sequence (Tuskan et al., 2006; Jansson and Douglas, 2007) offers a new model system for better understanding the adaptive mechanisms of woody plants in extreme environments.

In poplars, the transcriptomic mechanisms responding to abiotic stresses have been exploited in several species (Janz et al., 2010; Qiu et al., 2011; Chen et al., 2012a; Tang et al., 2013; Song et al., 2014; Zhang et al., 2014; Harfouche et al., 2014 review), and most of these studies have investigated long-term salt stress responses, such as *P. simonii* × *P. nigra* treated for 3, 6, and 9 days (Chen et al., 2012a), or from single-time point treatments, such as *P. simonii* treated for 6 h (Song et al., 2014). Moreover, most studies mainly focus on the leaves' responses to abiotic stresses, except for one study in *P. pruinosa* using calli (Zhang et al., 2014). Nonetheless, roots are the primary tissue exposed to salinity and are responsible for perceiving the stress signal under salt stress. Therefore, a transcriptome study of salt-stressed roots would be particularly useful for furthering the genetic improvement of *Populus* to this abiotic stress. To obtain insights into the initial perception mechanism in response to short-term high salinity stimuli in poplar roots, we examined gene expression changes over 24 h (0, 6, 12, and 24 h) and identified the salinity-specific regulatory networks, including ROS and hormones. This study would shed light on the mechanism of high salinity tolerance in poplar and provide a useful reference for further exploration in woody plants.

## Materials and methods

### Plant materials and experimental design

Chinese white poplar (*P. tomentosa*), one of the most widely planted poplar species with outstanding quality of wood, good resistances to adverse stresses in Northern China, was selected and cultured in the State Key Laboratory of Tree Genetic and Breeding (China). Briefly, the tissue-cultured seedlings of poplar were removed from the tissue culture containers after 8 weeks of cultivation and transferred to new containers in a gradually open environment to acclimatize. After 2 weeks of acclimation, the seedlings were treated with 200 mM NaCl for different time points (0, 6, 12, and 24 h). Three biological replicates were prepared for each time point. For the treated leaves, parts of them were used to detect H_2_O_2_, and parts of them were used to detect hormones. For the treated roots, parts of them were used to RNA-seq (two independent biological duplicates for sequencing in this study), and parts of them were used to quantitative real-time PCR (qRT-PCR). All the samples were frozen and stored in liquid nitrogen.

### RNA isolation, cDNA library construction, and illumina sequencing

RNA-seq libraries were constructed according to the manuals provided by Illumina and then sequenced on the HiSeq2000 platform. In brief, total RNAs were isolated from roots according to the instructions of the PLANT easy extraction protocol (BLKW, Beijing, China) and then treated with RNase-free DNase I (NEB, USA) to remove any contaminating genomic DNA. mRNAs were purified from total RNAs using poly-T oligo-attached magnetic beads, fragmented and used for cDNA synthesis with random hexamer primers and superscript II reverse transcriptase (Invitrogen, USA). After purification of the PCR products (AMPure XP system) and assessment of library quality on the Agilent Bioanalyzer 2100 system, four paired-end cDNA libraries were sequenced using the Illumina-Solexa sequencing platform.

### Reads mapping

Reference genome and gene model annotation files were downloaded directly from the poplar genome website (ftp://ftp.ncbi.nih.gov/genomes/PLANTS/Populus_trichocarpa/). An index of the reference genome was built using Bowtie v2.0.6 and single-end clean reads were aligned to the reference genome using TopHat v2.0.9 (Trapnell et al., 2009). Read counts were calculated using HTSeq (www.huber.embl.de/users/anders/HTSeq) and normalized to the RPKM value (reads per kilobase per million reads; Mortazavi et al., 2008) to obtain the relative expression levels.

### Statistical analysis and functional annotation

Principal component analysis (PCA) was performed using the R statistical environment (version 3.1.1). The R packages of DESeq (Anders and Huber, 2010) were used for pairwise gene expression comparisons between control (0 h) and treatment groups (6 vs. 0 h, 12 vs. 0 h, and 24 vs. 0 h). The significance of differentially expressed genes (DEGs) was identified with an “adjusted *p* ≤ 0.05” (in DESeq, the adjusted *p*-value considers multiple testing using the Benjamini-Hochberg method) and “|log2Ratio|≥1.” For GO enrichment and pathway analysis, all DEGs that were identified in all pairwise comparisons were mapped to terms in the Gene Ontology (GO) and Kyoto Encyclopedia of Genes and Genomes (KEGG), and significantly enriched terms were identified in comparison with the genome background.

Noted: In pairwise comparisons between control and treatment groups, firstly the Ratio of treatment group vs. control group was needed to calculation, and then log2Ratio as relative fold was done. So, when the read accounts of unigenes in control group were 0 (no expression for these unigenes under control), the values of Ratio (treatment vs. control) could not be operated. Therefore, in this condition we assigned the log2Ratio values to |13| as relative folds to be convenient to the following analysis, which was beyond the maximum values in log2 (treatment/control) of this study.

### Gene expression pattern analysis by clustering

In order to identify the up/down regulation pattern in the data, we used the Spearmean rank correlation distance as defined by:

$$\begin{array}{l} 1-\text{cor}\left(\text{xi},\text{xj}\right). \end{array}$$

Where cor(·) is the function to calculate Spearman correlation coefficient, xi and xj are the expression of two genes in the four time points.

To analyze the time course expression pattern of DEGs, K-means clustering was used to cluster the identified DEGs based on RPKM values obtained as described above. To obtain a more stringent result, we only retained genes with total sum of RPKM values greater than 12 from the four time points. In the K-means clustering, we used Hartigan and Wong (1979) algorithm, and maximum number of allowed iteration was set to 10. K-means cluster is a non-deterministic algorithm, namely, dependent on the initial status, the runs of the algorithm may resulted in different cluster assignment. Therefore, we run the algorithm 10 times with random initialization. Then, a ratio of “within-cluster sum of squares/between-cluster sum of square” (WSS/BSS ratio) was used to evaluate the quality of the cluster result. A low value of this criterion indicates a cluster assignment. Finally, we selected the clustering result that has the lowest WSS/BSS ratio and also has a clear four-cluster structure representing the slow/quick up/down regulated genes.

Additionally, we performed hierarchical clustering using the R statistical environment (version 3.1.0), which demonstrated very similar clustering trends, as shown in the Supplementary Table S8B, and the genes in each hierarchical clustering were then enriched using DAVID (https://david.ncifcrf.gov/gene2gene.jsp).

### Validation of DEGs with qRT-PCR

First strand cDNA was synthesized from 1.0 μg total RNA digested by DNase I by using the PrimeScript™ RT reagent Kit for RT-PCR (Takara, China), according to the manufacturer's instructions. The specific primers for poplar were synthesized by the Shanghai Sangon biotechnology company (Shanghai, China). qRT-PCR used a SYBR Premix ExTaq™ kit (Takara, China) in an ABI 7500. To quantify the relative expression level of the target genes, the poplar reference gene *Actin* (GenBank Accession No. AY261523.1/U60491) was used as the internal control. The *Actin* gene primer pairs were as follow: forward, 5′-CTCCATCATGAAATGCGATG-3′; reverse, 5′-TTGGGGCTAGTGCTGAGATT-3′ (Du et al., 2013a). Three independent biological replicates were performed for each selected gene (two time technical repeats per biological replicate).

### Histochemical localization of H_2_O_2_

H_2_O_2_ was detected *in situ* using 3,3-diaminobenzidine (DAB), as described previously (Shi et al., 2010). Briefly, the leaves of poplar from the above treatments were immersed in 10 mM 2-(N-morpholino) ethane sulfonic acid (MES; pH 6.5, 0.1% DAB) under vacuum for 5 min and incubated for 8 h in the dark at room temperature, before being placed in the light until a brown coloration appeared. After decoloring in boiling ethanol, the seedlings were photographed. The data shown were representative of three replicated experiments.

### Determination of related physiological indices

#### H_2_O_2_

The H_2_O_2_ contents were measured using a hydrogen peroxide assay kit (Beyotime Institute of Biotechnology, Beijing, China). H_2_O_2_ could oxidize Fe^2+^ to Fe^3+^, and then Fe^3+^ reacted with xylenol orange, leading to colorimetric reaction that could be detected using a microplate reader (SpectraMax Paradigm Multi-Mode Microplate Reader, USA). Briefly, samples were placed at room temperature for 30 min and measured immediately using the microplate reader at 560 nm. The concentration of H_2_O_2_ released was calculated from a standard concentration curve with three replicate experiments (two time technical repeats per biological replicate).

#### GSH and GSSG

The contents of GSH (reduced glutathione) and GSSG (oxidized glutathione) were measured using a GSH and GSSG assay kit (Beyotime Institute of Biotechnology), following the method of Anderson (1985). Briefly, total glutathione (GSH plus GSSG) was determined using a microplate reader (SpectraMax Paradigm Multi-Mode Microplate Reader, USA) at 412 nm for 25 min after precipitation with 0.1 M HCl, using glutathione reductase, 5,5-dithio-bis-(2-nitrobenzoic acid) (DTNB) and NADPH. GSSG was determined by the same method in the presence of GSH, whose content was calculated from the difference between total glutathione and GSSG. The levels of GSH and GSSG were expressed as mg/g protein, with three replicated experiments (two time technical repeats per biological replicate).

#### Ascorbate (AsA)

The AsA contents were measured as in Kampfenkel et al. (1995), based on the reduction of Fe^3+^ to Fe^2+^ by AsA and the spectrophotometric detection of the Fe^2+^ complex with 4,7-Diphenyl-1, 10-phenanthroline (bathophenanthroline, BP). Leaf samples were ground in liquid nitrogen and placed in test tubes with 500 μl 5% TCA. Supernatant was immediately assayed for AsA. AsA was determined using a microplate reader at 534 nm for 30 min using the following solutions: supernatant of samples (standard solutions of AsA), 5% (w/v) TCA, 0.4% (v/v) H_3_PO_4_ dissolved in ethanol, 0.5% (w/v) BP dissolved in ethanol, and 0.3% (w/v) FeCl_3_ dissolved in ethanol. The AsA contents were calculated with three replicated experiments (two time technical repeats per biological replicate).

#### ABA, GA, and IAA

The concentrations of indole acetic acid (IAA), abscisic acid (ABA), and gibberellic acid (GA) were determined using an HPLC (Agilent 1100 series) system following the method of Xu et al. (1998). Each sample (0.3 g) was homogenized in liquid nitrogen with 5 ml 80% methanol, and the homogenate was stirred overnight at 4°C. After centrifugation and filtration, the aqueous sample was then poured onto a PVP column to remove impurities. The sample was adjusted to pH 2.5 and partitioned against ethyl acetate. The sodium bicarbonate solution (containing the free IAA, ABA, and GA) was partitioned against ethyl acetate and dried three times. The residue was dissolved in a solution (0.5 mL of 3% methanol and 97% acetic acid), and applied onto a column (Agilent TC-C_18_, 250 mm × 4.6 mm, 5 μm). The IAA, ABA, and GA were determined at 267 nm, BW = 16 nm; reference wavelength = 360 nm, BW = 100 nm. Three replicated experiments were performed.

## Results

### Changes in H_2_O_2_ and ABA in poplar subjected to salt stress

**Figure 1:**
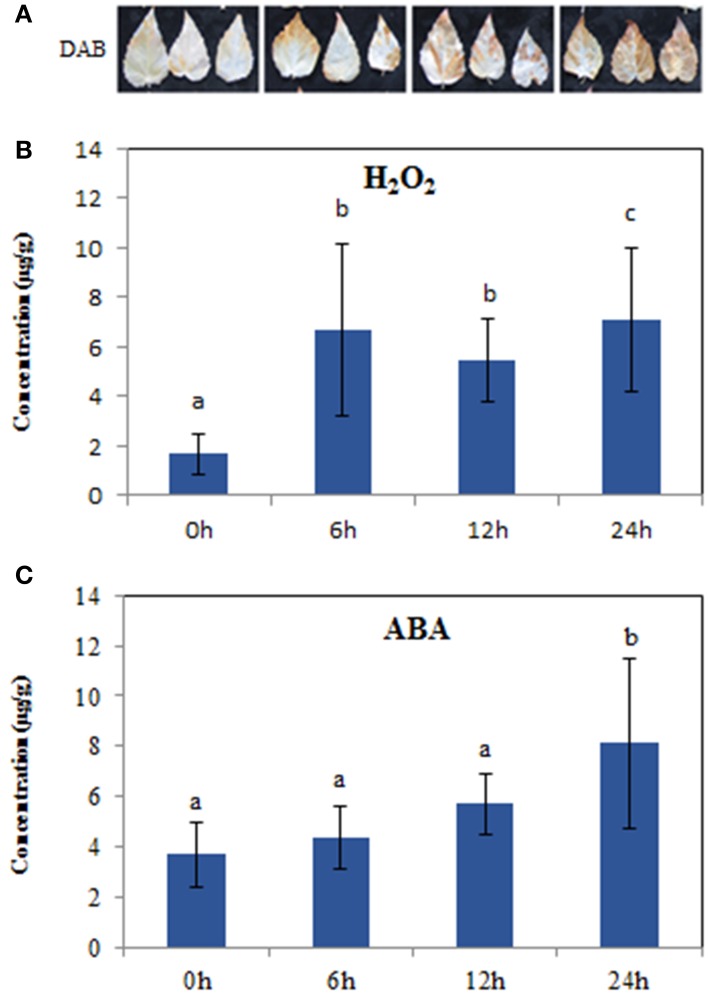
**Hydrogen peroxide and ABA levels in the leaves of 10-week seedlings of ***Populus tomentosa*** under 200 mM NaCl for 0, 6, 12, 24 h. (A)** DAB staining showed H_2_O_2_ accumulation *in situ*; **(B)** H_2_O_2_ concentration; **(C)** ABA concentration. Vertical bars (when indicated) represent the mean ± SD a representative of three separate experiments. Data were analyzed by ANOVA in the SPASS software. a, b, c indicate statistically significant differences (*P* < 0.05) for the designated time point.

To verify accumulation of H_2_O_2_ in response to salt stress, we employed 3, 3′-diaminobenzidine (DAB) staining to detect H_2_O_2_ *in situ*. A significant increase in H_2_O_2_ was observed at 6, 12, and 24 h compared to controls (0 h) (Figure 1A), and the change trend was similar to the determination of H_2_O_2_ content, which showed a statistically significant increase at 6 h (Figure 1B). The contents of ABA, an important stress hormone, showed an obvious increase at 24 h compared to controls (Figure 1C). Additionally, salt stress also induced the production of hormones GA and IAA (Supplementary Figure S1). Further comparison showed that the significant accumulation in H_2_O_2_ began at 6 h, while the increases in hormones were at 24 h, under the salt treatments of 6, 12, and 24 h (Figures 1B,C; Supplementary Figure S1). These findings suggested that these time points might show a substantial change in the expression of salt responsive genes, especially ROS/hormone-related genes. Therefore, we next used RNA-seq to profile the gene expression at each time point to deeply understand the short-term salt stress response in poplar.

### Overview of RNA-seq data of poplar subjected to salt stress

**Figure 2:**
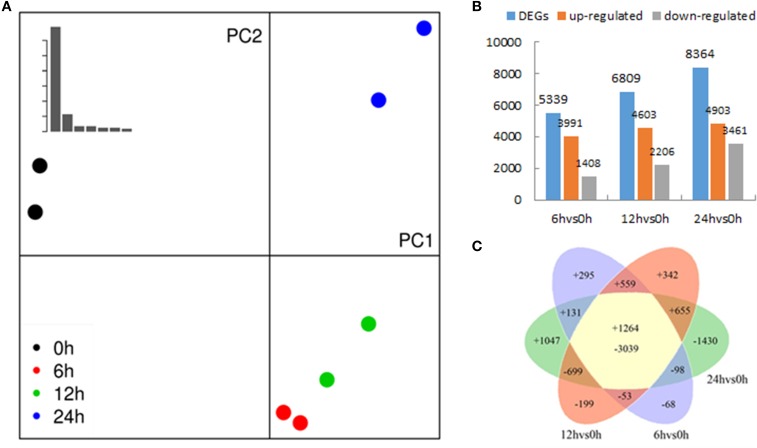
**Transcriptomes of *Populus tomentosa* under salt treatments detailed in Figure 1. (A)** Principal component analysis (PCA) of the RNA sequencing data. The eight samples (two biological replicates of 0, 6, 12, and 24 h) were projected on the first and second principal components (PC). The scree plot at the top left shows the Eigen values of PCs, which indicates that PC1 and PC2 account for >90% of the total variance in the data; different colors of dots indicate duplicates at different time points, and the control samples; treated sample are separated by PC1 (percentage of variance), whereas PC2 separates different time points; duplicates are projected together, which suggests that the duplicates are more similar. **(B)** Numbers of differentially expressed genes (DEGs). **(C)** Venn diagrams showing unique and shared DEGs between the salt treated transcriptomes compared with the control.

A summary of raw data is shown in Table 1 and the quantitative expression of genes (estimated by RPKM values) is shown in Table 2. A high correlation between biological replicates was observed (R^2^ > 0.94 for all four treatments (Supplementary Table S1), which indicated that the biological replicates were reliable in this study. To further access the data quality, PCA (Abdi and Williams, 2010) was performed. The results suggested that the PC, which represents the difference between the control (0 h) and treatment groups (6, 12, 24 h), captured most of the variance in the data. The samples from the time points could be separated by the second axis, which captured less variance in the data (Figure 2A). In addition, the biological replicates were projected closely in the space, which indicated a good correlation between replicates. Moreover, the mRNA expression data also suggested that the expression difference between control and treated groups are dramatically greater than that between different time points of treatment (Figure 2A).

**Table 1:** **Summary of the Illumina-Solexa DGE sequencing tags and their matches in the *Populus trichocarpa* genome**.

**Summary**	**0 h**	**6 h**	**12 h**	**24 h**
Total raw reads	7661489	7302935	8233693	7258612
Total clean reads	7644365	7287053	8220266	7248102
GC content	44.94%	44.77%	44.21%	45.14%
Total mapped reads	5527773	5350154	5288792	6120909
Total mapped reads/Total clean reads	72.16%	72%	68.01%	69.90%
Multiple mapped reads	392528	414091.5	515992.5	471650
Multiple mapped reads/Total clean reads	5.11%	5.7%	6.28%	6.5%
Uniquely mapped reads	5135275	4767960	5420976	4176384
Uniquely mapped reads/Total clean reads	67.04%	65.48%	65.95%	57.62%

*The treatments are as Figure 1*.

**Table 2:** **Percentages of gene different expression levels**.

**RPKM**	**0 h(%)**	**6 h(%)**	**12 h(%)**	**24 h(%)**
0~1	39.22	37.22	37.66	38.53
1~3	11.23	12.69	12.78	14.07
3~15	27.18	28.69	28.35	27.04
15~60	16.32	15.63	14.88	14.70
>60	6.06	6.09	5.84	5.67

*The treatments are as Figure 1*.

### Pairwise comparisons of transcriptome between control and salt treated roots

**Figure 3:**
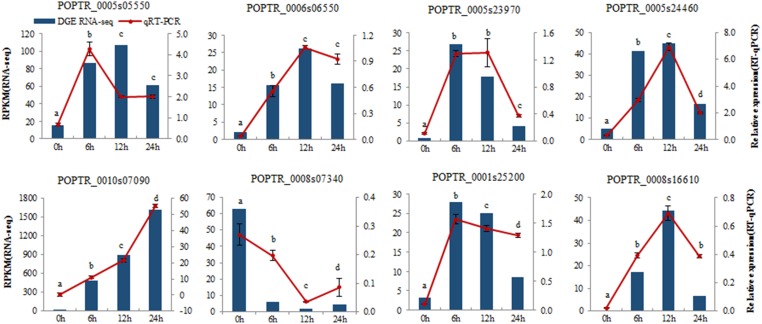
**Verification of eight selected DEGs by qRT-PCR**. Comparison of RNA-seq data (Blue bar) with qRT-PCR data (Red line). The normalized expression level (RPKM; reads per kilobase per million reads) of RNA-seq is indicated on the y-axis to the left. The relative qRT-PCR expression level is shown on the y-axis to the right. *Actin* was used as the internal control. Both methods show similar gene expression trends. Three biological replicates were performed. Data were analyzed by ANOVA in the SPASS software, and a, b, c, d indicate statistically significant differences (*P* < 0.05) for the designated time point.

To determine the gene expression changes resulting from salt treatment, DEGs between control samples (0 h) and each group of treated samples (6, 12, 24 h) were identified. The |log2Ratio| ≥1, not a higher fold value, was used as the threshold to screen the DEGs to get more information on genes potentially involved in the poplar salt response. Compared with controls (0 h), 3991, 4603, and 4903 genes were up regulated, and 1408, 2206, and 3461 genes were down regulated after exposure to salt stress for 6, 12, and 24 h, respectively. There were 1264 DEGs common to all three treatment stages compared with the untreated 0 h (Figures 2B,C; Supplementary Table S2). The upregulated genes accounted for 73.92, 67.60, and 58.62% of total DEGs at 6, 12, and 24 h, respectively. This suggested that the expressions of a large proportion of salt-responsive genes were induced at the initial stage of salt treatment, which would aid poplar to mount a rapid response to stressful signals. To validate the differential expression analysis by RNA-seq, eight genes were randomly selected from the identified DEGs for qRT-PCR analysis (Supplementary Table S3). The result showed a good correlation between the RPKM values and qRT-PCR results (Figure 3), which suggested the DEGs identified by two biological replicates were reliable at the expression level.

**Figure 4:**
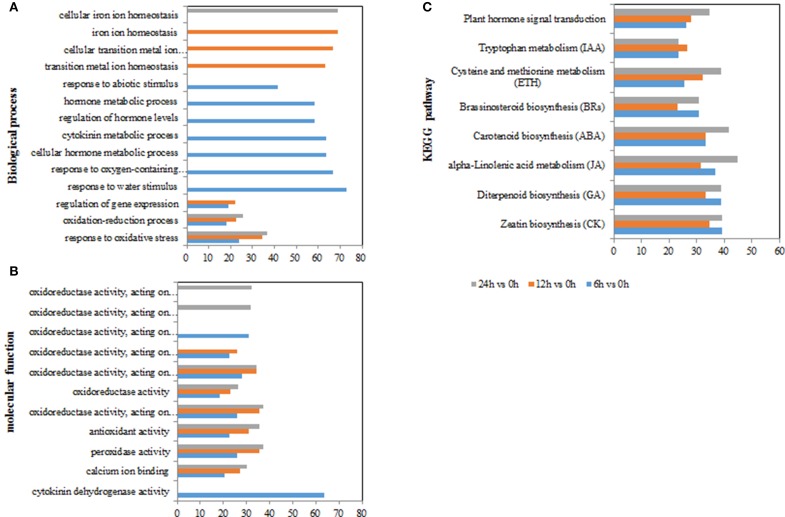
**GO terms and KEGG pathways involved in ROS and hormones production in *Populus tomentosa* under salt treatment**. **(A)** Biological process GO terms; **(B)** Molecular function GO terms; **(C)** KEGG pathways. The x-axis in **(A,B)** indicates the percentage of DEGs numbers vs. background gene numbers in each GO term; the x-axis in **(C)** indicates the percentage of DEGs numbers vs. background gene numbers in each KEGG pathway. The detailed information is shown in Supplementary Tables S4, S5 and Table 3.

To gain an insight into the functions of the DEGs, GO and KEGG pathway enrichment analysis was applied (using hypergeometric tests). GO annotation suggested biological processes and molecular function related to ROS functions were enriched among the DEGs at time points (e.g., “response to oxidative stress,” “oxidoreductase activity acting on peroxide as acceptor,” “peroxidase activity,” “antioxidant activity,” and “calcium ion binding”) (Figures 4A,B and Supplementary Tables S4A,B). In particular, biological processes related to hormones were only significantly enriched with DEGs at 6 h, including “cellular hormone metabolic processes,” “regulation of hormone levels,” “hormone metabolic process,” “response to abiotic stimulus” (Figure 4A; Supplementary Tables S4A,B). In addition, the molecular function term “oxidoreductase activity, acting on single donors with incorporation of molecular oxygen, incorporation of two atoms of oxygen” was enriched at 6 h, but the term of “oxidoreductase activity, acting on the CH-OH group of donors, NAD or NADP as acceptor” occurred specifically at 24 h (Supplementary Tables S4A,B). The results indicated that hormones and ROS metabolism play crucial roles in poplar's response to salt stress.

For the KEGG pathway enrichment analysis, 111, 115, and 118 pathways were categorized from the pairwise comparisons between 6 vs. 0 h, 12 vs. 0 h, and 24 vs. 0 h, respectively (Supplementary Table S5). Based on the ranks of the top 30 pathways from 6 vs. 0 h, the biosynthetic pathways of seven hormones were enriched in the DEGs (“carotenoid biosynthesis,” “zeatin biosynthesis,” “diterpenoid biosynthesis,” “brassinosteroid biosynthesis,” “cysteine and methionine metabolism,” “alpha-linolenic acid metabolism,” and “tryptophan metabolism”), as was one pathway for “plant hormone signal transduction” (Figure 4C; Table 3). However, we observed that the gene numbers of four hormone synthetic pathways remained relatively stable under the salt treatments of 6, 12, and 24 h; whereas those in the ABA and ethylene pathways increased with increasing salt-treatment times. Additionally, 55.6% of the genes associated with the pathway “steroid biosynthesis” had higher expression at 24 h, although the figure was only 18.5% at 6 h. Another two pathways “glutathione metabolism” and “oxidative phosphorylation,” which are involved in ROS metabolism, also showed a more than 2-fold increase in the percentage DEGs at 24 h than at 6 h (Supplementary Table S5). These results demonstrated that salt treatment significantly induced the expression of genes involved in hormones and ROS production.

**Table 3:** **Top 30 KEGG pathways based on the percentage of DEGs in 6 vs. 0 h**.

**Term**	**Total**	**6 vs. 0 h**		**12 vs. 0 h**		**24 vs. 0 h**	
		**DEGs**	**%**	**DEGs**	**%**	**DEGs**	**%**
Flavonoid biosynthesis	35	22	62.9	24	68.6	25	71.4
Selenocompound metabolism	20	9	45.0	13	65.0	12	60.0
Linoleic acid metabolism	18	8	44.4	8	44.4	9	50.0
Taurine and hypotaurine metabolism	15	6	40.0	7	46.7	3	20.0
Zeatin biosynthesis (CK)	23	9	39.1	8	34.8	9	39.1
Diterpenoid biosynthesis (GA)	18	7	38.9	6	33.3	7	38.9
Plant-pathogen interaction	138	53	38.4	54	39.1	61	44.2
alpha-Linolenic acid metabolism (JA)	38	14	36.8	12	31.6	17	44.7
Stilbenoid, diarylheptanoid, and gingerol biosynthesis	14	5	35.7	5	35.7	6	42.9
Biosynthesis of unsaturated fatty acids	37	13	35.1	11	29.7	16	43.2
Ribosome	406	142	35.0	143	35.2	203	50.0
Phenylpropanoid biosynthesis	118	40	33.9	53	44.9	56	47.5
Carotenoid biosynthesis (ABA)	36	12	33.3	12	33.3	15	41.7
Vitamin B6 metabolism	9	3	33.3	3	33.3	3	33.3
C5-Branched dibasic acid metabolism	6	2	33.3	2	33.3	2	33.3
Phenylalanine metabolism	105	34	32.4	44	41.9	47	44.8
ABC transporters	26	8	30.8	9	34.6	8	30.8
Brassinosteroid biosynthesis (BRs)	13	4	30.8	3	23.1	4	30.8
Nitrogen metabolism	46	14	30.4	14	30.4	16	34.8
Riboflavin metabolism	10	3	30.0	3	30.0	3	30.0
Plant hormone signal transduction	319	84	26.3	89	27.9	110	34.5
Isoquinoline alkaloid biosynthesis	27	7	25.9	5	18.5	7	25.9
Cysteine and methionine metabolism (ETH)	106	27	25.5	34	32.1	41	38.7
Alanine, aspartate and glutamate metabolism	57	14	24.6	17	29.8	23	40.4
Tryptophan metabolism (IAA)	34	8	23.5	9	26.5	8	23.5
Starch and sucrose metabolism	179	41	22.9	50	27.9	60	33.5
Galactose metabolism	53	12	22.6	12	22.6	13	24.5
Tropane, piperidine and pyridine alkaloid biosynthesis	31	7	22.6	7	22.6	10	32.3
Fatty acid elongation	27	6	22.2	6	22.2	7	25.9
Cyanoamino acid metabolism	37	8	21.6	9	24.3	11	29.7

*Hormone biosynthetic and signal pathways were marked in gray background*.

### Analysis of gene temporal expression pattern

**Figure 5:**
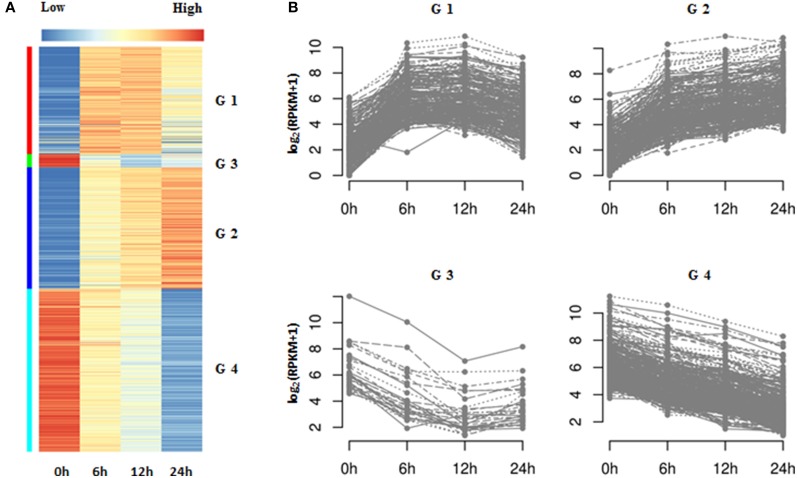
**K-means clustering of DEGs in *Populus tomentosa* under salt treatment. (A)** Heat map; **(B)** Line chart. Four subgroups of DEGs were classified further based on the total RPKM value ≥12. The detailed information is shown in Supplementary Tables S6, S7.

To further explore the temporal expression patterns in the data, we applied k-means clustering to the identified DEGs. In this analysis, we only included DEGs whose total sum of RPKM values were greater than 12 from among the four categories, which represented a more stringent threshold. K-means clustering demonstrated that the 940 qualifying genes could be categorized into four groups (referred as G1, G2, G3, and G4), comprising 249, 282, 31, and 378 genes respectively (Figures 5A,B; Supplementary Table S7). G1 and G2 genes were characterized as upregulated by salt treatment. The expressions of G1 genes increased in a time dependent manner, especially at 6 h; whereas that of G2 genes was continuously upregulated at 6, 12, and 24 h. The other two groups, G3 and G4, comprised genes that were downregulated by salt treatment. The expressions of G4 genes had a clear negative correlation with prolonged salt treatment, while those of G3 genes decreased rapidly at 6 or 12 h, and then remained stable. Therefore, we assumed that both G1 and G3 genes are genes rapidly induced in response to salt treatment, whereas G2 and G4 consist of slow (time-dependent) responsive genes in this process. Hierarchical clustering produced four group genes with similar expression trends to those of K-means clustering, except for a few changes of gene numbers among them (Supplementary Tables S7, S8A,B).

Further functional analysis of the four groups of genes suggested that G1 genes were significantly enriched with processes of transcriptional factor activities (e.g., “transcription factor activity,” “transcription regulator activity”) and response to stimulus, particular for the stimuli of hormones (e.g., “response to organic substance,” “response to endogenous stimulus,” “response to hormone stimulus,” “regulation of transcription,” and “cellular response to hormone stimulus”) (Supplementary Table S8C). However, the G4 genes were related to the metabolism of cell wall organization (e.g., “cell wall organization,” “external encapsulating structure organization,” and “cell wall modification”) and hydrogen peroxide (e.g., “response to hydrogen peroxide,” “hydrogen peroxide catabolic process,” “response to reactive oxygen species,” and “response to oxidative stress”) (Supplementary Table S8). Interestingly, there were no GO terms significantly associated with G2 and G3 genes. A further manual examination of these genes suggested that G2 genes mostly participated in transcription regulation, calcium signal transduction and the production and scavenging of H_2_O_2_; while G3 contained genes involved in hormone biosynthesis (e.g., 1-aminocyclopropane-1-carboxylate oxidase 1 gene; *ACO1*) and hormone signal transduction (e.g., ABA receptor *PYL4,6*) (Supplementary Tables S7, S8A).

Both the pairwise comparisons and K-means/hierarchical clustering demonstrated that the genes related to ROS and hormones are significantly affected during the early stage of salt stress in poplar roots. Hence, we further explored the DEGs involved in the metabolism of ROS and hormones, and their signal pathways systematically.

### DEGs involved in H_2_O_2_ production and scavenging

**Figure 6:**
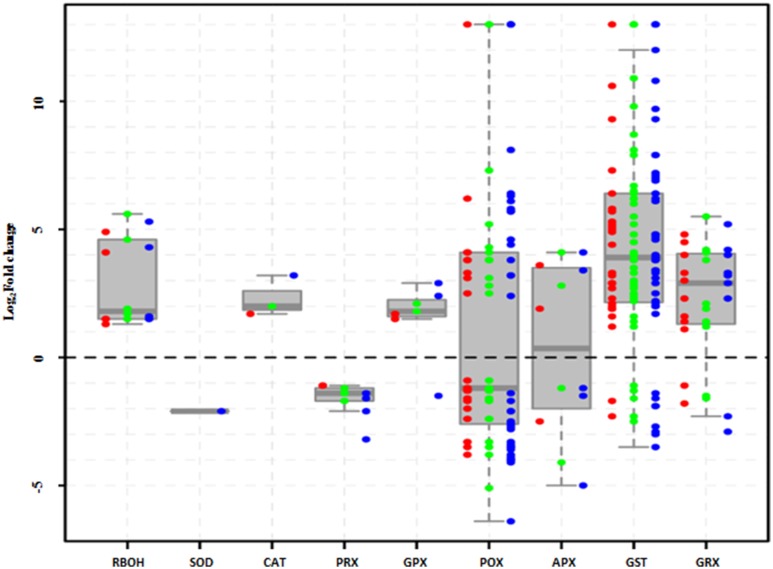
**Expression of H_2_O_2_ production- and scavenging-related genes in *Populus tomentosa* under salt treatment**. Red, green, and blue dots represent the values of each gene of Log_2_ (read counts 6 h/0 h), Log_2_ (read counts 12 h/0 h), and Log_2_ (read counts 24 h/0 h) for the DEGs. The detailed information is shown in Supplementary Table S9.

During abiotic stresses, ROS accumulation depends greatly on the balance between ROS production and ROS scavenging (Miller et al., 2010). In this study, the H_2_O_2_-producing genes, *RBOHs* (respiratory burst oxidase homologs), were significantly upregulated, while H_2_O_2_-scavenging genes showed different expression patterns under salt stress (Figure 6). For example, *SOD* and *PRX*s were inhibited, whereas *CAT* and *GPX*s were enhanced; however, four other kinds of H_2_O_2_-scavenging genes were included among the upregulated and downregulated groups.

NaCl stress induced the upregulation of *RBOH* gene family, which are one of the main sources of H_2_O_2_ generation and include *RBOHA, C, D*, at all the time points (Figure 6; Supplementary Table S9). Simultaneously, ROS scavenging systems were markedly influenced, showing different expression patterns under salt treatment (Figure 6; Supplementary Table S9). For example, the peroxidase (POD) gene family comprises 41 members, including 36 Class III *POX*, three *GPXs* and two *APXs*, and approximately half of them were either upregulated or downregulated. Most of the 46 glutathione S-transferase (GST) genes were significantly upregulated (Figure 6; Supplementary Table S9). Meanwhile, peroxiredoxins (PRXs) and glutaredoxins (GRXs) genes showed completely opposite expression patterns; three *PRX* genes were totally inhibited, whereas most of 11*GRX* genes continuously increased under salt treatments. However, among catalases (CATs) and superoxide dismutases (SODs), only one member was affected under salt stress. These results suggested that the enzymatic pathways of *POD, GST*, and *GRX* gene families play particularly important roles in protecting poplar against oxidative damage under salt stress.

### DEG genes involved in hormone biosynthetic pathways

**Figure 7:**
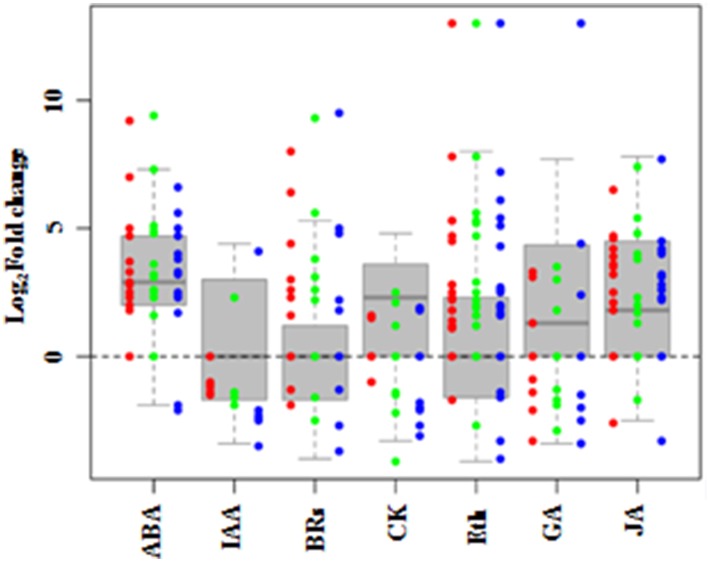
**DEGs involved in hormone synthesis in *Populus tomentosa* under salt treatment**. Red, green, and blue dots represent the values of each gene of Log_2_ (read counts 6 h/0 h), Log_2_ (read counts 12 h/0 h) and Log_2_ (read counts 24 h/0 h) for the DEG genes. The seven hormones are abscisic acid (ABA), auxin (IAA), cytokinin (CK), Gibberellin (GA), ethylene (ETH), Brassinosteroids (BRs), and Jasmonic acid (JA). The detailed information is shown in Supplementary Table S10.

To examine systematically the effect of salt stress on the pathways of hormone biosynthesis, the seven related KEGG pathways (biosynthesis of ABA, ethylene, auxin, cytokinin, GA, BRs, and JA; Table 3; Supplementary Table S10) were further examined manually (Supplementary Table S11). Most of the genes in the ABA, cytokinin, GA, and JA biosynthetic pathways showed increased expression, while genes involved in auxin, BRs, and ethylene biosynthesis did not show uniform expression trends (Figure 7).

Under salt stress, 15 genes in the ABA biosynthetic pathway were identified as DEGs, including phytoene synthase (*PSY*), lycopene beta cyclase (*LCYB*), beta-carotene hydroxylase 2 (*CH2*), zeaxanthin epoxidase (*ABA2*), violaxanthin de-epoxidase (*VDE*), 9-cis-epoxycarotenoid dioxygenase 1 (*NCED1*), and abscisic acid 8 hydroxylase 1, 2, and 4 (*ABAH1, 2, 4*). Most of them were significantly induced, except for *LCYB* and *VDE* at 24 h. Moreover, the expression of *NCED1*, an important biosynthetic gene, increased to 9-fold at 6 h compared with its level at 0 h, before gradually decreasing. By contrast, the expressions of four *ABAHs*, key ABA catabolic genes, maintained stably higher expression under salt stress (Supplementary Table S10). Twenty-two genes involved in the ethylene biosynthetic pathway were detected as DEGs (Supplementary Table S10). Among the genes for precursor synthesis, only one of five S-adenosyl methionine (SAM) synthase genes was continuously upregulated; the other four were significantly downregulated. All five 1-aminocyclopropane-1-carboxylate (ACC) synthase genes (*ACS*s) were significantly induced, but only three of seven ACC oxidase genes (*ACO*s) were induced, while other four were inhibited gradually. These results showed that the key regulatory components of the biosynthetic pathways of two stress hormones, ABA and ethylene, were changed significantly during poplar's response to salt stress.

BRs and JA also play essential roles in plant responses to abiotic stress. The expressions of nine genes involved in BRs biosynthetic pathways changed under salt stress (Figure 7). Two of three cytochrome P450 (CYP) genes participating in BRs biosynthesis, *CYP724B1* and *CYP90A1*, increased significantly; while *CYP85A* had no obvious expression before 12 h, but decreased by 3-fold at 24 h. The two genes encoding steroid 5-alpha-reductase (DET2), a major rate-limiting enzyme in BRs biosynthesis, showed different expression trends, one displayed a continuous increase to more than 5-fold expression levels compared with 0 h, whereas the other one was downregulated (Supplementary Table S10). For the JA biosynthetic pathway, most of the 19 genes were markedly induced by salt (Figure 7), especially the allene oxide synthase (AOS) genes, which are the first enzyme in the branch pathway leading to JA biosynthesis. Two *AOSs* displayed continuous increases to more than 7-fold expression from 6 h, and the other two gradually decreased (Supplementary Table S10). These findings indicated that BRs and JA also actively participate in the stress response of poplar.

GA, auxin and cytokinin, are widely considered to participate in regulating plant development, but many of their genes showed increased transcript abundances in response to salt (Figure 7). Interestingly, seven gibberellin 2-beta-dioxygenase 2 (GA2ox2) genes showed three temporal expression patterns: two *GA2ox2*s were expressed only at 6 h and 24 h, respectively; while the other three maintained higher expression levels under the salt treatments of 6, 12, and 24 h, compared with 0 h (Supplementary Table S10). The results suggested that these three classes of phytohormones are involved in the salt stress response of poplar.

### DEGs involved in hormone signal pathways

We further mined systematically the DEGs involved in the signal transduction of seven hormones, which corresponded to hormone synthetic pathways, from the KEGG pathway “plant hormone signal transduction” (Table 3; Supplementary Tables S8, S11).

**Figure 8:**
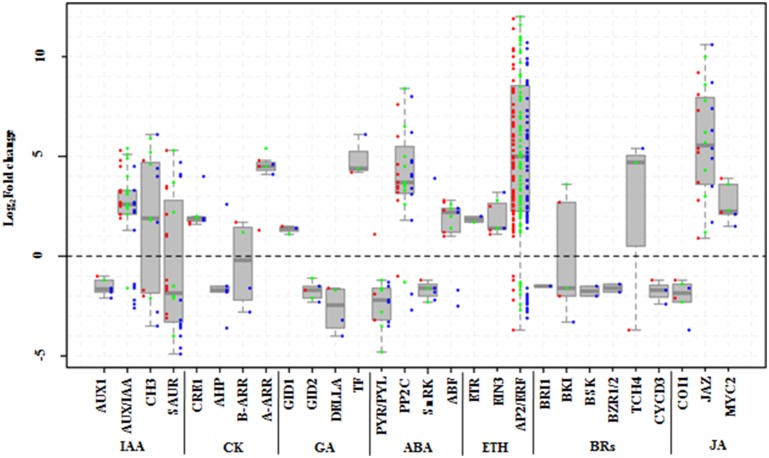
**DEGs involved in signal pathways of seven hormones in *Populus tomentosa* under salt treatment**. Red, green, and blue dots represent the values of each gene of Log_2_ (read counts 6 h/0 h), Log_2_ (read counts 12 h/0 h), and Log_2_ (read counts 24 h/0 h) for the DEG genes. The seven hormones are auxin (IAA), cytokinin (CK), Gibberellin (GA), abscisic acid (ABA), ethylene (ETH), Brassinosteroids (BRs), and Jasmonic acid (JA). The detailed information is shown in Supplementary Table S11.

In the ABA signaling pathway, the expression of nine *PYR/PYL*s, 12 *PP2C*s, five *SnRK2*s, and seven *ABF*s were changed under salt stress (Figure 8). Most of *PYR/PYL*s, including *PYR/PYL1, 2, 4, 6, and 9*, were significantly downregulated (except *PYR/PYL4* member at 6 h). Similarly, all the five *SnRK2*s were repressed. While most of *PP2C*s had remarkable increases, even one *PP2C75* was de novo induced, except for the decrease of two *PP2C16*s. Likewise, five of seven *ABF*s expression increased, another two just decreased at 24 h under salt stress (Supplementary Table S11). For ethylene signal pathway, only one *ETR* and three *EIN3*s were induced. Surprisingly, 99 *ERF1/2*s were found to be changed by NaCl treatments, and most of them (84.8%) were up regulated including12 *ERF1/2*s induced initially by salt stress (Figure 8; Supplementary Table S11).

Besides, both BRs and JA hormone signal pathways also participate widely in regulating stress responses in plants. Six components of the BR signaling pathway, *BRI1, BKI, BSK, BZR1/2, TCH4*, and *CYCD3*, were detected under salt stress (Figure 8). Of these, the expressions of one of two *BKI* members increased; and *TCH4* expression decreased dramatically at 6 h, while it increased remarkably at subsequent time points; and others (*BSK, BZR1/2*) were mainly repressed at 24 h (Supplementary Table S11). In the JA signal pathway, however, except for the expressions of two *COI1*s, which are main node genes, showed stable decreases, the expressions of nine *JAZ*s (*TIFY* family), and two *MYC2*s increased significantly. In particular, most of the *JAZ*s' expressions continuously increased (Supplementary Table S11).

In the GA signal pathways, the *GID1, GID2, DELLA*, and *TF/PIF5* genes showed similar trends to the genes of the cytokinin signal pathway, although one member of both *GID2* and *DELLA* began to be repressed at 6 h (Supplementary Table S11).

## Discussion

In plants, the response to salinity takes places in two phases: a rapid, osmotic phase that inhibits the growth of young leaves, and a slow, ionic phase that accelerates the senescence of mature leaves (Munns and Tester, 2008). When salt stress occurs, the stress signal is first perceived by receptors, which results in generation of many secondary signal molecules, such as Ca^2+^ (Steinhorst and Kudla, 2013), ROS (Gill and Tuteja, 2010; Miller et al., 2010) and hormones (Cui et al., 2011; Bhaskara et al., 2012; Du et al., 2013b; Merchante et al., 2013; Chater et al., 2014). The signal molecules are then transduced into the nucleus, which further activates numerous stress responsive genes to induce salt stress tolerance (Chen et al., 2012b). In this work, we used RNA sequencing to explore the time course of the response mechanism in poplar under salt stress. Our study showed that 73.92% of the DEGs were upregulated at 6 h, and then decreased to 67.60 and 58.62% at 12 and 24 h, respectively (Figure 2B), which indicated that more genes were induced or activated efficiently at the initial stage of treatment to cope with high salinity in *P. tomentosa*.

Given the importance of ROS, ABA and other hormones in plant responses to abiotic stress (Pang and Wang, 2008; Bhaskara et al., 2012; Steffens et al., 2013; Chater et al., 2014; Ruiz-Sola et al., 2014), in the DEG analysis we particularly focused on the ROS and hormone metabolism, and related signaling pathways. Accumulation of ROS involves two mechanisms: ROS generation and ROS scavenging. Further functional analysis of related genes by GO enrichment revealed that mechanisms involved in transcriptional activation and hormone signaling are induced at the 6 h time point. However, cell wall organization, ROS homeostasis and certain peroxidase genes are continually changed in the entire time-scale of the experiment. This suggested that oxidative stress and hormones might be activated over different time periods in response to salt stress.

In response to salt stress, the fast (at 6 h) generation of ROS (Figures 1A,B) may be achieved by activation of ROS-producing genes, such as *RBOHs* (Figure 6). However, plants have evolved a series of mechanisms to maintain the homeostasis of ROS (Miller et al., 2010). In this study, we found that most of the *GPX, GST*, and *GRX* genes were expressed at higher levels under salt stress (Figure 6). The enzymes encoded by these genes play important roles in the reaction of peroxide detoxification by catalyzing GSH to GSSG (Marí et al., 2009). The involvement of this process was confirmed by the determination of GSH and GSSG contents (Supplementary Figures S2A,B), which showed that the GSH contents decreased dramatically, while the GSSG contents increased significantly. Likewise, the increase in the AsA contents (Supplementary Figures S1A,B) would also benefit ROS scavenging. However, prolonged stress exposure (to 24 h or more) potentially destroys ROS homeostasis in plants, which leads to the dramatic accumulation of H_2_O_2_ (Miller et al., 2010; Steffens et al., 2013). This observation is supported by the DAB staining and H_2_O_2_ measurement (Figures 1A,B), which demonstrated that the accumulation of H_2_O_2_ increased sharply at 24 h (Figures 1A,B). The functional analysis of the DEGs suggested the involvement of peroxidase related functions and cell wall organization (Figures 4, 6; Supplementary Tables S8, S9). Our study identified 41 genes of the *POD* superfamily from poplar whose expressions were altered under salt stress, including *POX, GPX*. Half of them were upregulated under salt stress, whereas Ren et al. (2013) found that more than 70 *POD*s were affected by salt stress (Cosio and Dunand, 2009). In the poplar genome, 93 genes encode POD proteins (Ren et al., 2013), while 73 *POD*s exist in *Arabidopsis* (Cosio and Dunand, 2009). PODs are plant-specific enzymes involved in lignin formation, the cross-linking of cell wall components, the removal of H_2_O_2_, the oxidation of toxic reductants and defense against biotic stresses (Ren et al., 2013). Additionally, among these enzymatic pathways, only one member of the *SOD* and *CAT* gene family, respectively, were detected as differentially expressed, although these two types of enzymes are known ROS scavengers (Gill and Tuteja, 2010; Miller et al., 2010; Song et al., 2014). This Cu/Zn *SOD* expression was only inhibited at 24 h; the gene family of *SOD*, however, includes three classes, based on their active site metal cofactors (Fe, Mn, or Cu and Zn), suggesting that SODs may not be the main scavenger of ROS under short-term salt stress in poplar.

Meanwhile, many studies have also shown that salt stress-induced ROS accumulation could be a mechanism to protect plants rather than cause damage, at least at the initial stage (Mittler et al., 2004; Pang and Wang, 2008; Miller et al., 2010). In tomato, both BR and ABA can increase the expression of *RBOH1*, which further induces the accumulation of H_2_O_2_ (Zhou et al., 2014). In this study, ABA contents could maintain a certain level at the initial treatment (Figure 1C), which might benefit to induce *RBOH* expression (Zhou et al., 2014). The respiratory burst NADPH oxidases (RBOHs) are the main producers of signal transduction-associated ROS in cells under stress processes and promote the production of H_2_O_2_ (Miller et al., 2010; Steffens et al., 2013). Our transcriptomic data demonstrated that only one *RBOHC* in five upregulated *RBOH* members was expressed at 12 h under salt treatment and then disappeared (Supplementary Table S9), which might be related to BRs, because BRs can induce a rapid and transient H_2_O_2_ production via NADPH oxidase, which in turn triggers increased ABA biosynthesis, leading to further increases in H_2_O_2_ accumulation (Zhou et al., 2014). We also noted that the other four *RBOH*s had stable expressions (Supplementary Table S9) and the accumulation of H_2_O_2_ was maintained at a higher level (Figures 1A,B). Moreover, the accumulation of H_2_O_2_ triggered by ABA could activate Ca^2+^ channels involved in stomatal closure (Pei et al., 2000). Our study also found that many genes were induced in the Ca^2+^ signal pathway (Supplementary Table S12).

In the presence of ABA, PYR/RCARs, as ABA receptors, interact with PP2Cs and inhibit phosphatase activity, allowing SnRK2 activation and phosphorylation of target proteins to control ABI5, ABF, and RBOH gene expression (Cutler et al., 2010; Hubbard et al., 2010; Peleg and Blumwald, 2011). Accordingly, we observed that five members of *RBOHA, B*, and *C* were significantly induced under salt stress (Figure 6; Supplementary Table S9), which might be related to ABA signaling. Correspondingly, a rapid and massive H_2_O_2_ accumulation was detected at 6 h (Figures 1A,B). Previous studies proved that the upregulation of *PSY, LCYB, BCH2, ZEP*, and *NCED* contributes to ABA synthesis (Peleg and Blumwald, 2011; Ruiz-Sola et al., 2014). Our results also showed that all ABA biosynthetic genes (*PSY BCH2, ZEP NCED*) were activated under salt stress (Supplementary Table S10), but the ABA contents did not show a significant increase at the initial stage (Figure 1C). This might be related to the high expression of four *ABAH4/CYP707A* genes (Supplementary Table S10), which are key genes of ABA degradation to control ABA level (Matakiadis et al., 2009). However, the upstream genes of the BRs biosynthetic pathway, *STE1/DWF7* were expressed a low level, and *DWF5* and *DWF1*, which are involved in synthesizing the campesterol of BRs precursor (Chung and Choe, 2013) were inhibited at 24 h. Moreover, the last-step gene, *DWARF/CYP85A*, was also downregulated under salt stress (Supplementary Table S10), which is almost opposite to the expression of DEGs in the ABA biosynthetic pathways. Similarly, almost all DEGs involved in the BRs signaling pathway were down regulated under salt stress. These results might also explain the antagonistic relationship of gene expression levels between BRs and ABA displayed in several physiological responses (Zhou et al., 2014).

Similarly, H_2_O_2_ also influences other hormone signaling pathways. Previous studies indicated that H_2_O_2_ induced stomatal closure, requiring the ethylene receptor *ETR1*, whose expression was upregulated in poplar under NaCl stress (Supplementary Table S11), implying a link between H_2_O_2_ and ethylene signal transduction (Desikan et al., 2005). Likewise, H_2_O_2_ treatment could enhance the expression of *HvGA20ox1*, which is implicated in GA synthesis during the germination of barley seeds (Bahin et al., 2011). This suggested that H_2_O_2_ could be involved in dormancy alleviation through activation of GA signaling and synthesis rather than repression. Our study, however, showed that two GA biosynthetic genes *GA20ox2*s were downregulated or had transiently low expression, whose defective expression in rice can result in a shortened culm with improved resistance (Spielmeyer et al., 2002). Hence, the *GA20ox2* expression repressed by salt stress might benefit the enhancement of salinity resistance.

**Figure 9:**
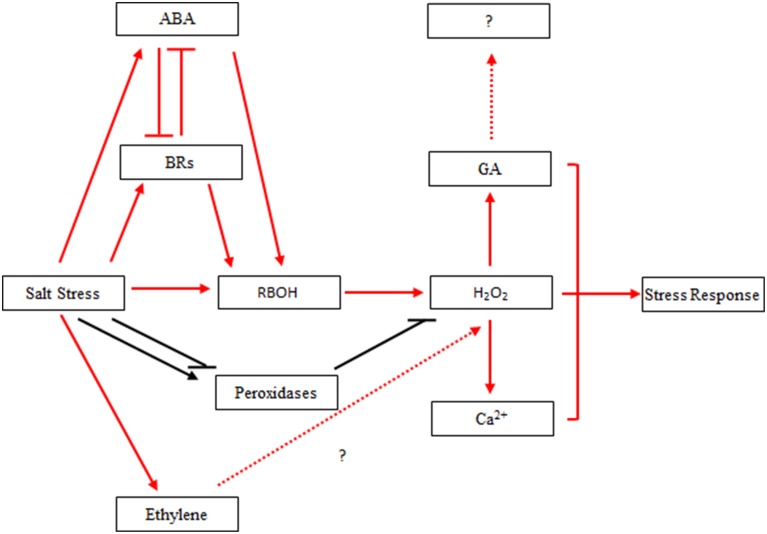
**Hypothetical interaction network between H_2_O_2_ and hormones in *Populus tomentosa* under salt stress**. Red arrow: salt stress induced hormone biosynthesis and signal transduction in the early part of the treatment, which leads to H_2_O_2_ accumulation; Black arrow: salt stress induced the continuous activation of H_2_O_2_ homeostasis under 24-h salt stress; Blunt arrow: negative regulation.

Combined with previous studies, our results implied that there might be a connection among H_2_O_2_ and hormone signal molecules involved in ABA and other hormones signal transductions to mediate salinity stress in poplar. However, the exact nature of these interactions in poplar (Figure 9) requires further investigation.

In conclusion, this study showed that short-term high salt treatment induced significantly the production in H_2_O_2_ and ABA in the Chinese white poplar (*P. tomentosa*) seedlings; moreover, the accumulation of H_2_O_2_ was much greater than that of ABA at the initial stage of salt stress. The dynamic transcriptome revealed that 5339, 6809, and 8364 of DEGs were identified at 6, 12, and 24 h after salt treatment. The percentage of DEGs upregulated at 6 h was higher than those at the latter two time points. Furthermore, the DEGs related to ROS and hormones were significantly involved in the response to salt stress, especially at the initial stage of salt treated poplar roots, which may benefit poplar's rapid response to cope with high salinity damage. Based on these findings, the crosstalk between H_2_O_2_ and ABA or other hormones will be further elucidated in future poplar studies, which could be used as a basis for improving salt stress tolerance in poplar species by genetic manipulation, and also be very useful for understanding of the molecular adaptation of other tree species to salinity stress.

## Deposited data

The RNA-seq datasets by using Illumina-Solexa platform are available from the NCBI Sequence Read Archive database (SRA; http://www.ncbi.nlm.nih.gov/sra) under project number accession SRP057808. The cDNA libraries obtained from the controls (0 h) and samples of 10-week poplar seedlings exposed to salt-stress for 6, 12, 24 h with two biological replicates, respectively.

## Author contributions

Manuscript draft: SS, CM, and LZ; Analyzing data: SS, CM, LZ, YM, and TC; Experiment: LZ, JM, XZ, EC, JJ, and ND, LC; Conception and supervision of the research: SS and ZJ.

### Conflict of interest statement

The authors declare that the research was conducted in the absence of any commercial or financial relationships that could be construed as a potential conflict of interest.

## Supplementary material

Supplementary Figure S1 **Concentration of GA and IAA in the leaves of 10-week seedlings of *Populus tomentosa* under 200 mM NaCl for 0, 6, 12, 24 h**. **(A)** GA; **(B)** IAA. Vertical bars represent the mean ± SD of three replicated experiments and the variances are marked above the vertical bars. The data were analyzed using ANOVA in the SPASS software. a and b indicate statistically significant differences (*P* < 0.05) for the designated time point.

Supplementary Figure S2 **Concentration of GSH, GSSG and Ascorbate (AsA) in the leaves of 10-week seedlings of *Populus tomentosa* under 200 mM NaCl for 0, 6, 12, 24 h. (A)** GSH; **(B)** GSSG; **(C)** AsA. Vertical bars represent the mean ± SD of three biological replicates (two time technical repeats per biological replicate) and the variances are marked above the vertical bars. The data were analyzed using ANOVA in the SPASS software. a, b, c and d indicate statistically significant differences (*P* < 0.05) for the designated time point.

Supplementary Table S1 **Pearson correlation analysis of the RPKM values of root samples in poplar under salt stress**.

Supplementary Table S2 **Read counts for differentially expressed genes under salt stress**.

Supplementary Table S3 **Primers for qRT-PCR**.

Supplementary Table S4 **GO enrichment of differentially expressed genes**.

Supplementary Table S5 **KEGG pathway enrichment of differentially expressed genes**.

Supplementary Table S6 **Total differentially expressed genes based on combined RPKM values**.

Supplementary Table S7 **Differentially expressed genes for K-means clustering**.

Supplementary Table S8 **Differentially expressed genes for Hierarchical clustering including DEGs, figure, and David analysis**.

Supplementary Table S9 **Differentially expressed genes for H_2_O_2_ production and scavenging**.

Supplementary Table S10 **Differentially expressed genes for hormone synthesis**.

Supplementary Table S11 **Differentially expressed genes for hormone signal pathways**.

Supplementary Table S12 **Differentially expressed genes for Ca^2+^ related genes under salt stress**.
