# Supplementary figures and images for: Transcriptomic analysis reveals importance of ROS and phytohormones in response to short-term salinity stress in Populus tomentosa
Source: Front Plant Sci. 2015 Sep 15;6:678. doi: 10.3389/fpls.2015.00678 (PMC4569970; doi:10.3389/fpls.2015.00678)

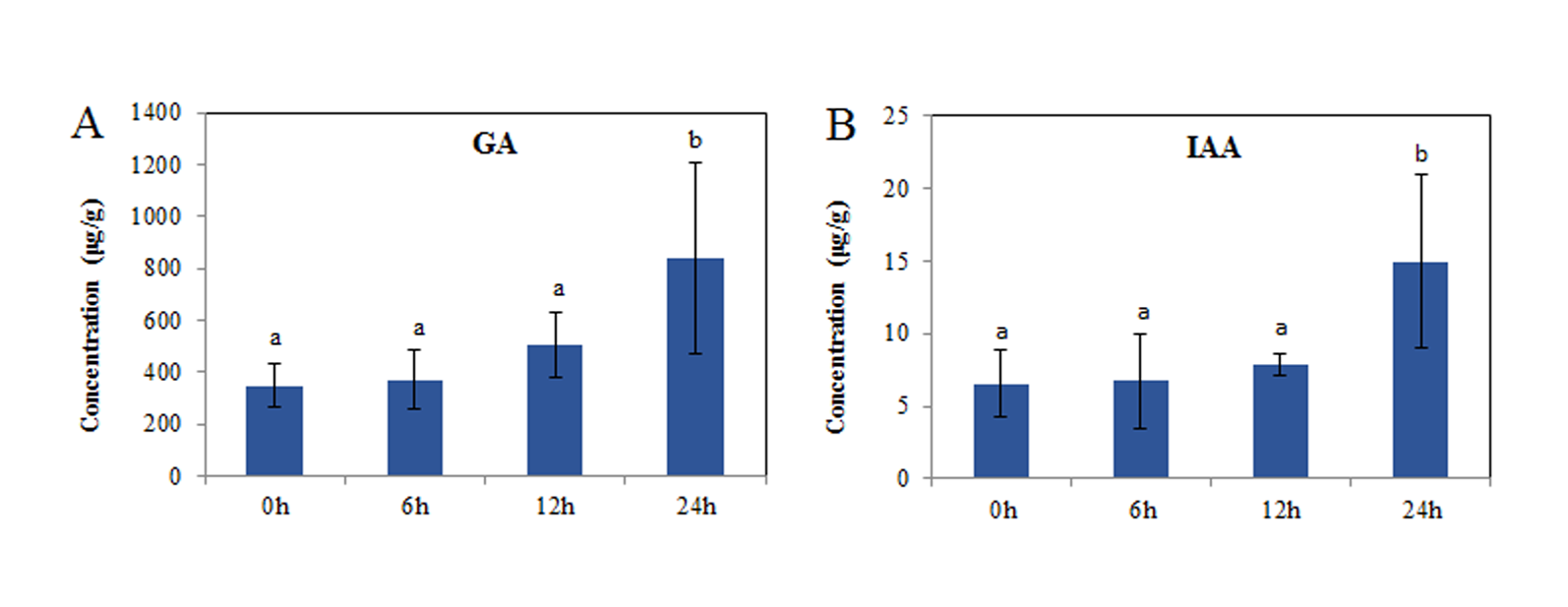

Supplement: Supplementary Figure S1 — Concentration of GA and IAA in the leaves of 10-week seedlings of Populus tomentosa under 200 mM NaCl for 0, 6, 12, 24 h. (A) GA; (B) IAA. Vertical bars represent the mean ± SD of three replicated experiments and the variances are marked above the vertical bars. The data were analyzed using ANOVA in the SPASS software. a and b indicate statistically significant differences (P < 0.05) for the designated time point. [file Image1.TIF]

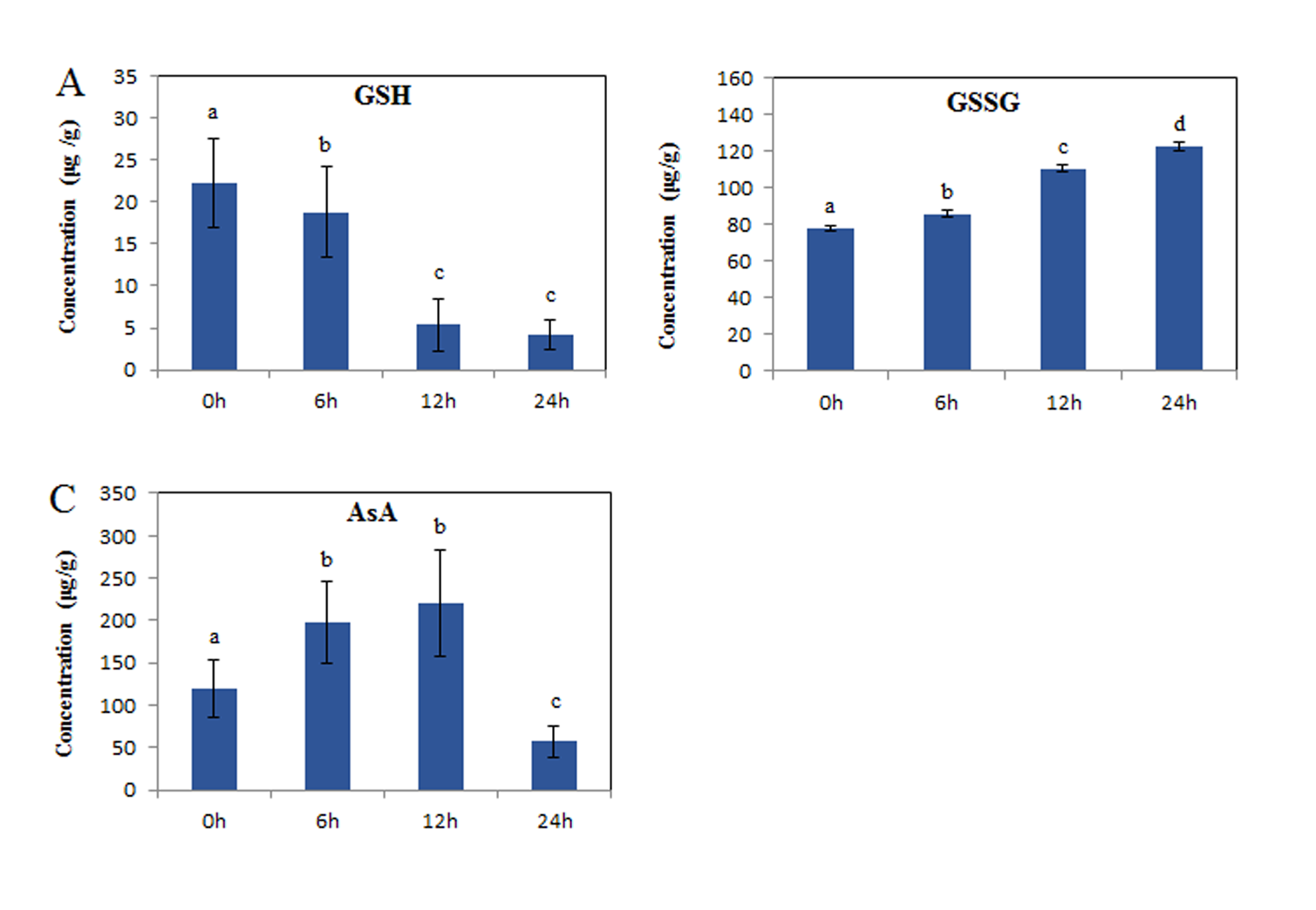

Supplement: Supplementary Figure S2 — Concentration of GSH, GSSG and Ascorbate (AsA) in the leaves of 10-week seedlings of Populus tomentosa under 200 mM NaCl for 0, 6, 12, 24 h. (A) GSH; (B) GSSG; (C) AsA. Vertical bars represent the mean ± SD of three biological replicates (two time technical repeats per biological replicate) and the variances are marked above the vertical bars. The data were analyzed using ANOVA in the SPASS software. a, b, c and d indicate statistically significant differences (P < 0.05) for the designated time point. [file Image2.TIF]
