# Supplementary material for: Transcriptomic analysis reveals importance of ROS and phytohormones in response to short-term salinity stress in Populus tomentosa
Source: Front Plant Sci. 2015 Sep 15;6:678. doi: 10.3389/fpls.2015.00678 (PMC4569970; doi:10.3389/fpls.2015.00678)
Supplement: Supplementary Table S3 — Primers for qRT-PCR. [file Table3.DOCX]

**Supplementary data table S3.** Primer sequences used for real-time PCR analysis.

| **Transcript ID** | **Primer sequence** |
| --- | --- |
| POPTR_0010s07090 | AAGCCAGTTTGCGAGTCCA |
|  | GACCCAGAAACGAGCCAGA |
| POPTR_0005s23970 | TTGCCTGGAAGTGATGAGC |
|  | CCATCCCACTACTTGTGCC |
| POPTR_0006s06550 | CCTGGGTTCATATTCGTGGTT |
|  | TCCTCGTTTGGCTGGTTATTC |
| POPTR_0005s05550 | GCTGAAGCTGCTGGACTATACGT |
|  | TCAGAAGGCTCACCAACTTTGA |
| POPTR_0008s07340 | TGTCGTCGAATCATACGTTGTG |
|  | ATCTGGTTATTCCGTGCCTTC |
| POPTR_0008s16610 | AGGCCACTCAAGAAACGTCAAA |
|  | CATGCCTTGTGGAATCCCTTAT |
| POPTR_0005s24460 | CATCAGGGTTTGGCTAGGGAC |
|  | GGCGAACACCCATCTTCACAT |
| POPTR_0001s25200 | GTAGCGATTCACCCTTCATTCT |
|  | TCTCCTTACACTTTACTGCCACAT |
